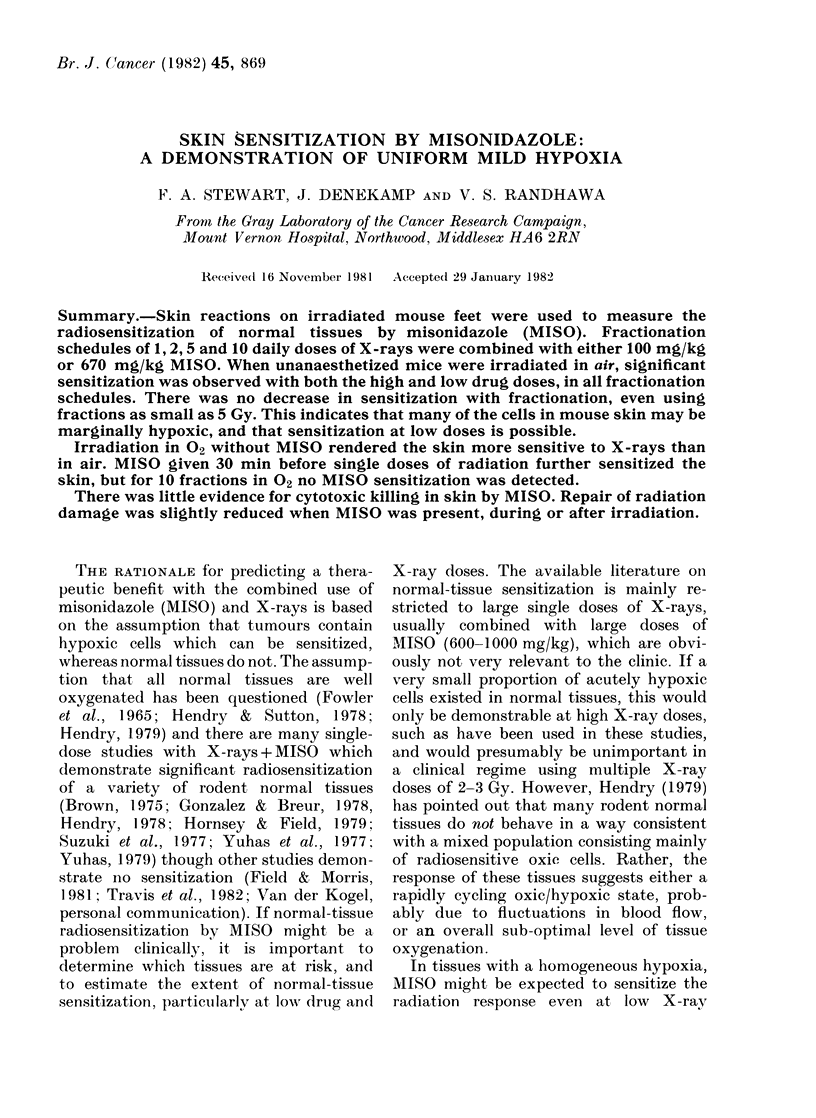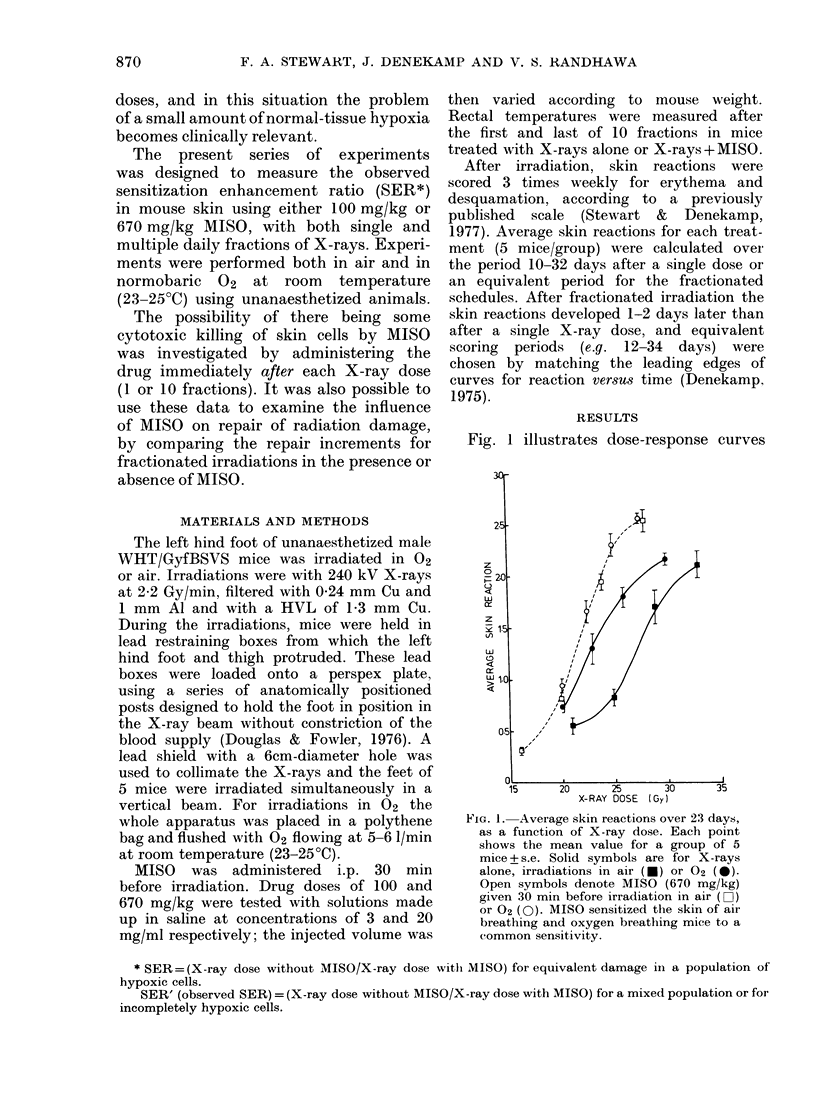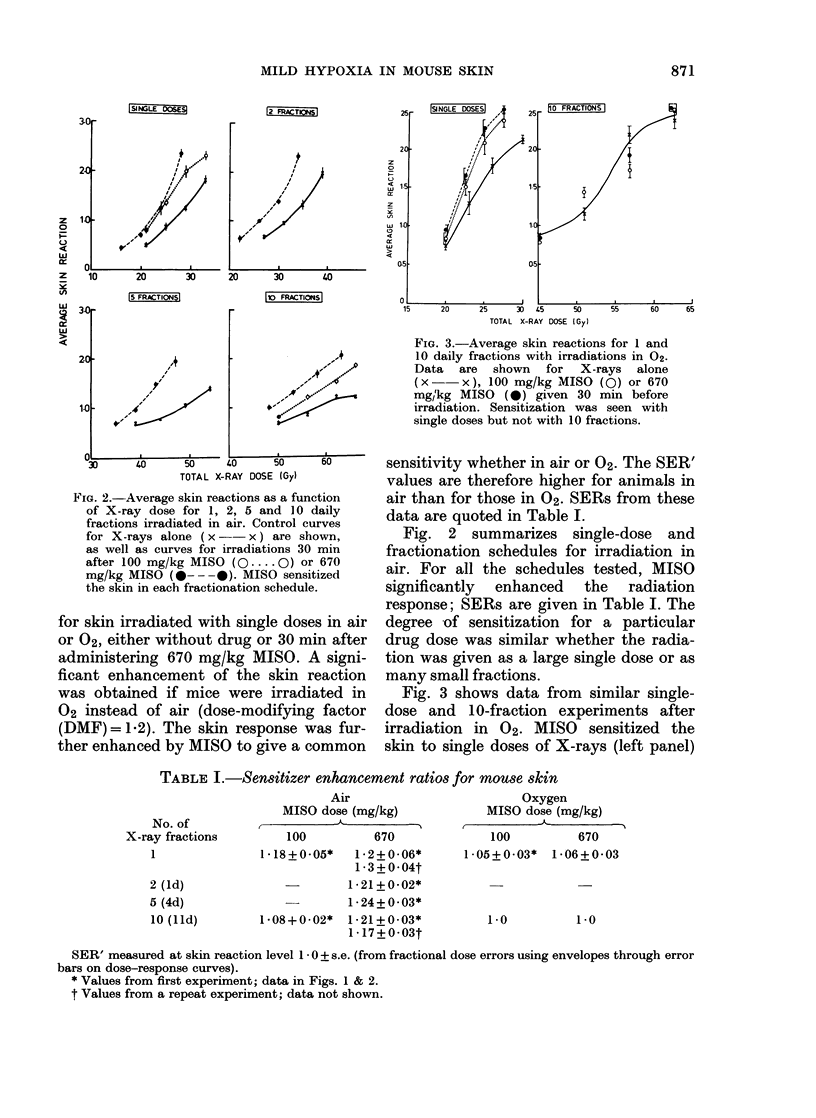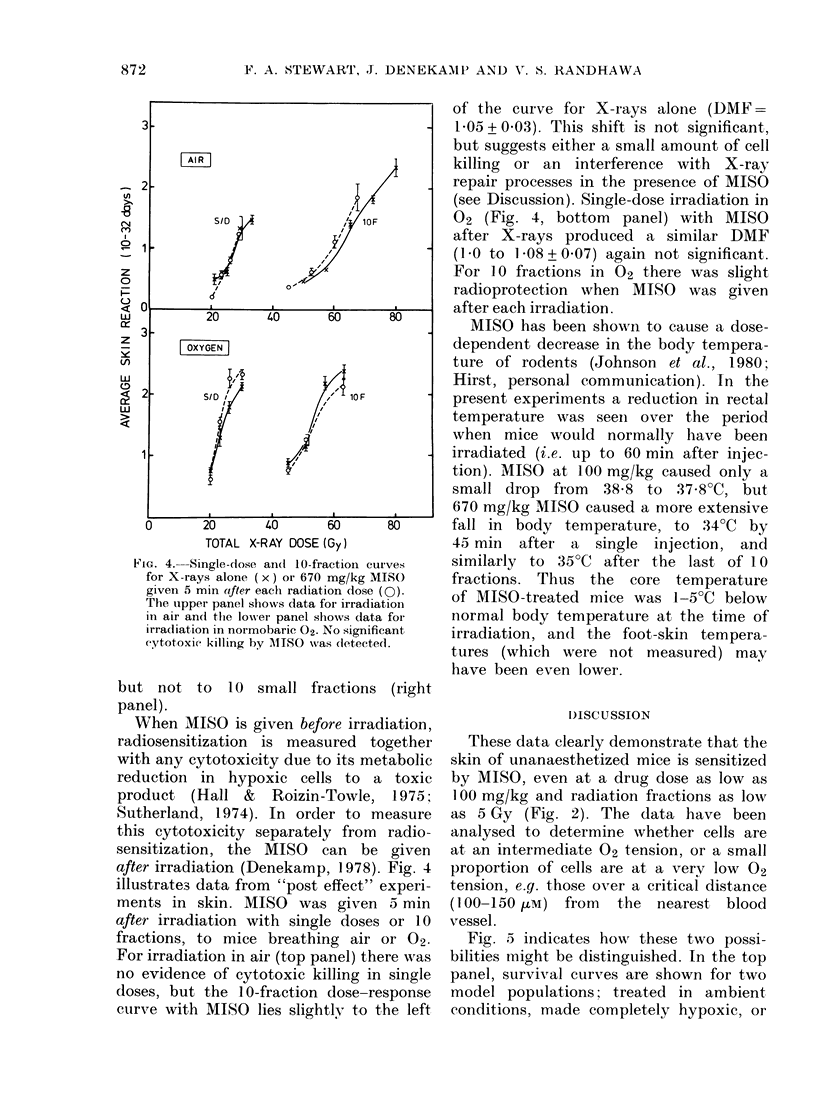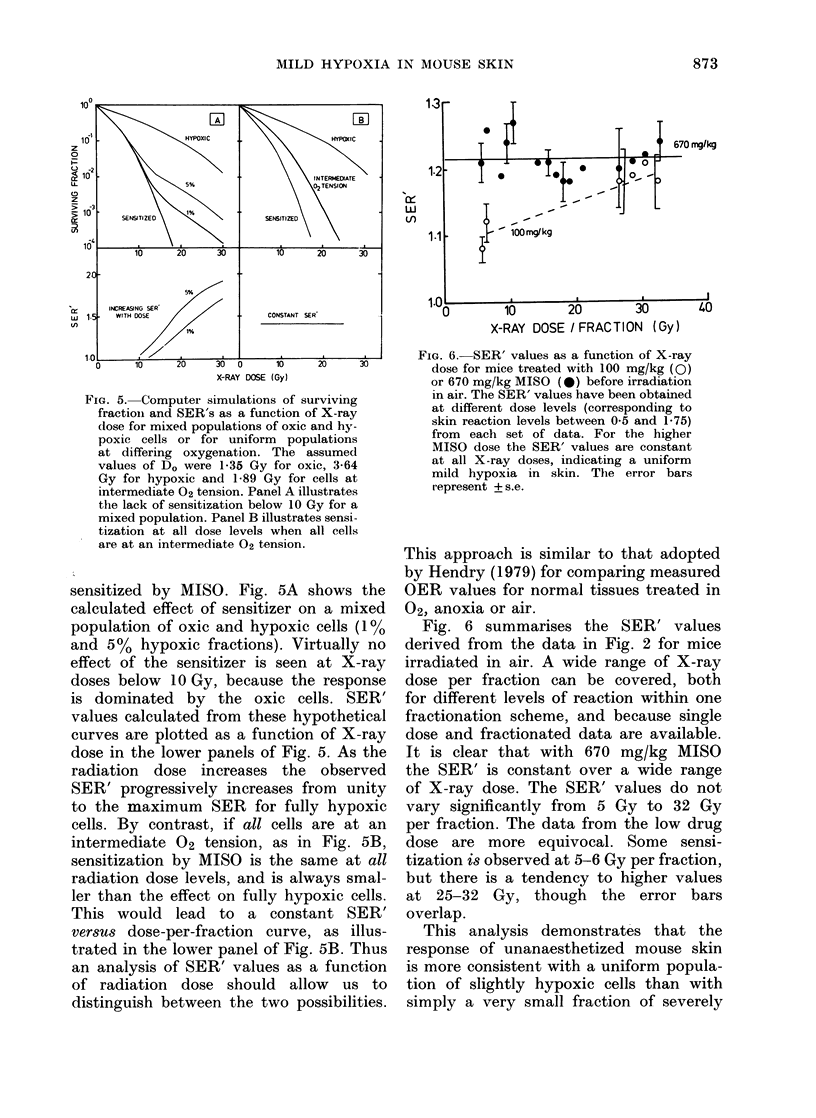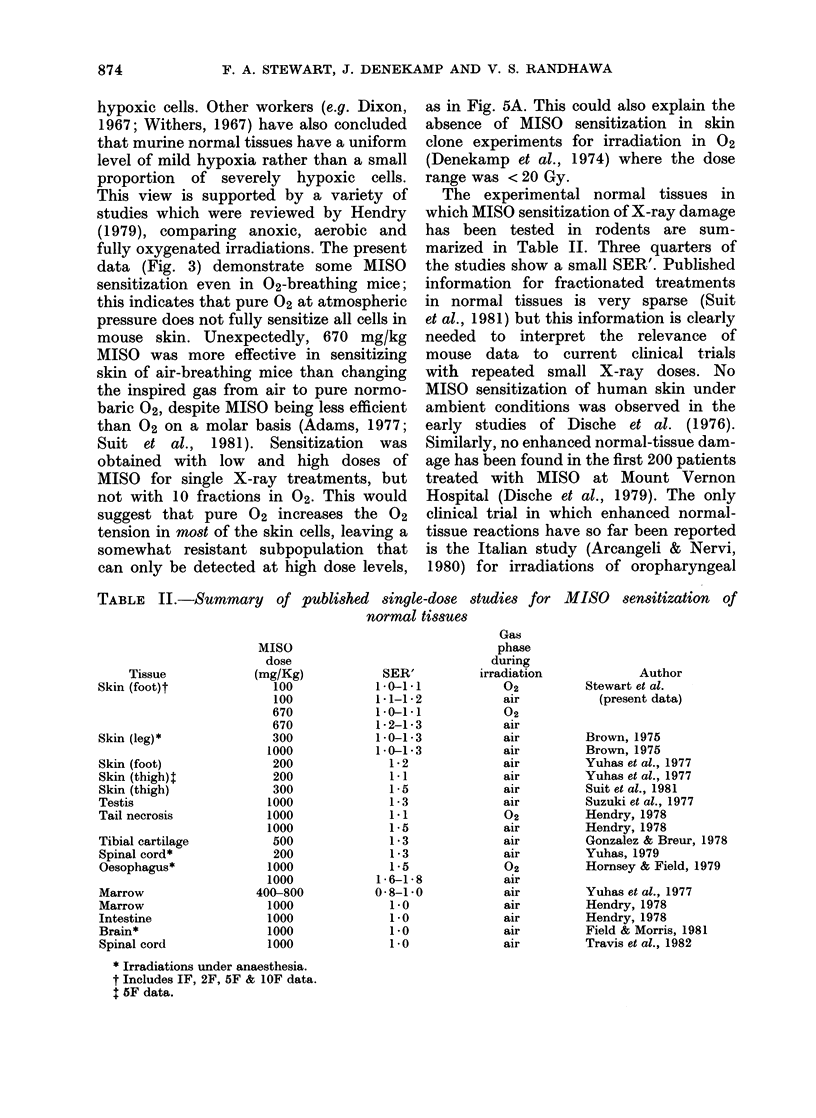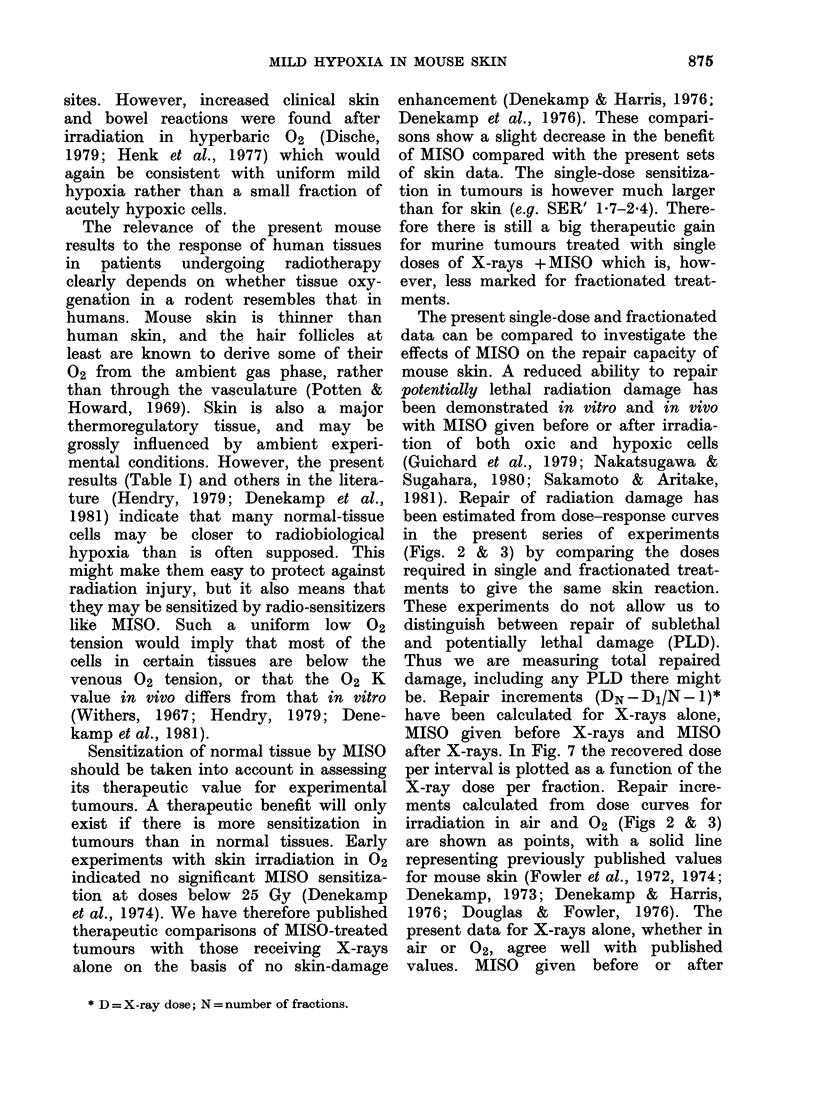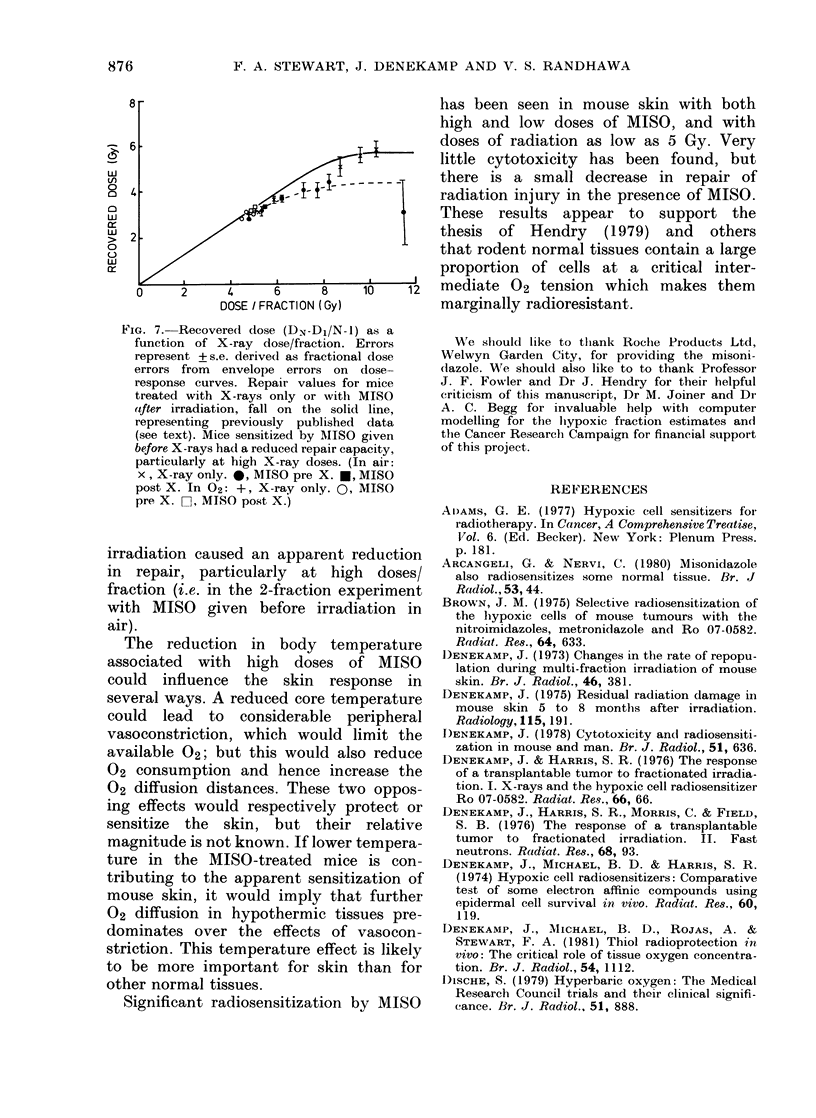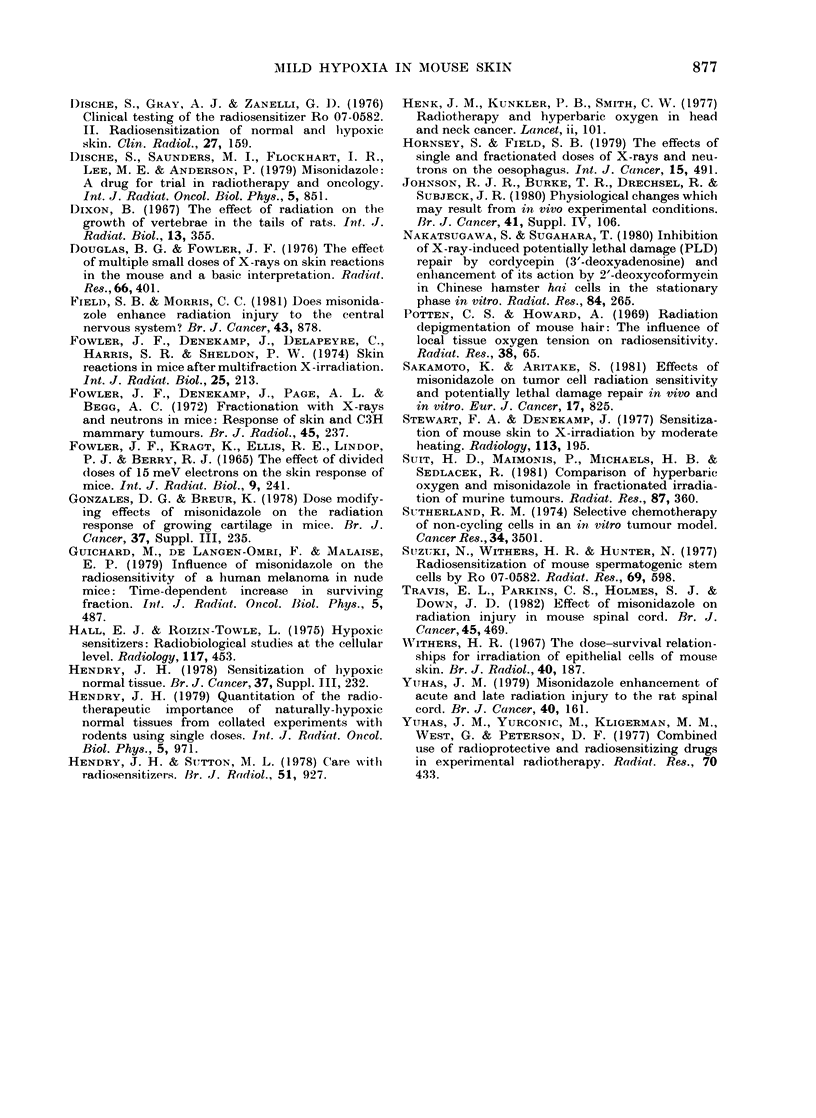# Skin sensitization by misonidazole: a demonstration of uniform mild hypoxia.

**DOI:** 10.1038/bjc.1982.139

**Published:** 1982-06

**Authors:** F. A. Stewart, J. Denekamp, V. S. Randhawa

## Abstract

Skin reactions on irradiated mouse feet were used to measure the radiosensitization of normal tissues by misonidazole (MISO). Fractionation schedules of 1, 2, 5 and 10 daily doses of X-rays were combined with either 100 mg/kg or 670 mg/kg MISO. When unanaesthetized mice were irradiated in air, significant sensitization was observed with both the high and low drug doses, in all fractionation schedules. There was no decrease in sensitization with fractionation, even using fractions as small as 5 Gy. This indicates that many of the cells in mouse skin may be marginally hypoxic, and that sensitization at low doses is possible. Irradiation in O2 without MISO rendered the skin more sensitive to X-rays than in air. MISO given 30 min before single doses of radiation further sensitized the skin, but for 10 fractions in O2 no MISO sensitization was detected. There was little evidence for cytotoxic killing in skin by MISO. Repair of radiation damage was slightly reduced when MISO was present, during or after irradiation.


Br. J. Cancer (1982) **45**, 869

### SKIN SENSITIZATION BY MISONIDAZOLE: A DEMONSTRATION OF UNIFORM MILD HYPOXIA

F. A. STEWART, J. DENEKAMP AND V. S. RANDHAWA

*From the Gray Laboratory of the Cancer Research Campaign, Mount Vernon Hospital, Northwood, Middlesex HA6 2RN*

Received 16 November 1981 Accepted 29 January 1982

**Summary.—Skin reactions on irradiated mouse feet were used to measure the radiosensitization of normal tissues by misonidazole (MISO). Fractionation schedules of 1, 2, 5 and 10 daily doses of X-rays were combined with either 100 mg/kg or 670 mg/kg MISO. When unanaesthetized mice were irradiated in *air*, significant sensitization was observed with both the high and low drug doses, in all fractionation schedules. There was no decrease in sensitization with fractionation, even using fractions as small as 5 Gy. This indicates that many of the cells in mouse skin may be marginally hypoxic, and that sensitization at low doses is possible.**

**Irradiation in $O_2$ without MISO rendered the skin more sensitive to X-rays than in air. MISO given 30 min before single doses of radiation further sensitized the skin, but for 10 fractions in $O_2$ no MISO sensitization was detected.**

**There was little evidence for cytotoxic killing in skin by MISO. Repair of radiation damage was slightly reduced when MISO was present, during or after irradiation.**

THE RATIONALE for predicting a therapeutic benefit with the combined use of misonidazole (MISO) and X-rays is based on the assumption that tumours contain hypoxic cells which can be sensitized, whereas normal tissues do not. The assumption that all normal tissues are well oxygenated has been questioned (Fowler *et al.*, 1965; Hendry & Sutton, 1978; Hendry, 1979) and there are many single-dose studies with X-rays+MISO which demonstrate significant radiosensitization of a variety of rodent normal tissues (Brown, 1975; Gonzalez & Breur, 1978, Hendry, 1978; Hornsey & Field, 1979; Suzuki *et al.*, 1977; Yuhas *et al.*, 1977; Yuhas, 1979) though other studies demonstrate no sensitization (Field & Morris, 1981; Travis *et al.*, 1982; Van der Kogel, personal communication). If normal-tissue radiosensitization by MISO might be a problem clinically, it is important to determine which tissues are at risk, and to estimate the extent of normal-tissue sensitization, particularly at low drug and X-ray doses. The available literature on normal-tissue sensitization is mainly restricted to large single doses of X-rays, usually combined with large doses of MISO (600–1000 mg/kg), which are obviously not very relevant to the clinic. If a very small proportion of acutely hypoxic cells existed in normal tissues, this would only be demonstrable at high X-ray doses, such as have been used in these studies, and would presumably be unimportant in a clinical regime using multiple X-ray doses of 2–3 Gy. However, Hendry (1979) has pointed out that many rodent normal tissues do *not* behave in a way consistent with a mixed population consisting mainly of radiosensitive oxic cells. Rather, the response of these tissues suggests either a rapidly cycling oxic/hypoxic state, probably due to fluctuations in blood flow, or an overall sub-optimal level of tissue oxygenation.

In tissues with a homogeneous hypoxia, MISO might be expected to sensitize the radiation response even at low X-ray doses, and in this situation the problem of a small amount of normal-tissue hypoxia becomes clinically relevant.

The present series of experiments was designed to measure the observed sensitization enhancement ratio (SER*) in mouse skin using either 100 mg/kg or 670 mg/kg MISO, with both single and multiple daily fractions of X-rays. Experiments were performed both in air and in normobaric $O_2$ at room temperature (23–25°C) using unanaesthetized animals.

The possibility of there being some cytotoxic killing of skin cells by MISO was investigated by administering the drug immediately *after* each X-ray dose (1 or 10 fractions). It was also possible to use these data to examine the influence of MISO on repair of radiation damage, by comparing the repair increments for fractionated irradiations in the presence or absence of MISO.

### MATERIALS AND METHODS

The left hind foot of unanaesthetized male WHT/GyfBSVS mice was irradiated in $O_2$ or air. Irradiations were with 240 kV X-rays at 2·2 Gy/min, filtered with 0·24 mm Cu and 1 mm Al and with a HVL of 1·3 mm Cu. During the irradiations, mice were held in lead restraining boxes from which the left hind foot and thigh protruded. These lead boxes were loaded onto a perspex plate, using a series of anatomically positioned posts designed to hold the foot in position in the X-ray beam without constriction of the blood supply (Douglas & Fowler, 1976). A lead shield with a 6cm-diameter hole was used to collimate the X-rays and the feet of 5 mice were irradiated simultaneously in a vertical beam. For irradiations in $O_2$ the whole apparatus was placed in a polythene bag and flushed with $O_2$ flowing at 5–6 l/min at room temperature (23–25°C).

MISO was administered i.p. 30 min before irradiation. Drug doses of 100 and 670 mg/kg were tested with solutions made up in saline at concentrations of 3 and 20 mg/ml respectively; the injected volume was then varied according to mouse weight. Rectal temperatures were measured after the first and last of 10 fractions in mice treated with X-rays alone or X-rays + MISO.

After irradiation, skin reactions were scored 3 times weekly for erythema and desquamation, according to a previously published scale (Stewart & Denekamp, 1977). Average skin reactions for each treatment (5 mice/group) were calculated over the period 10–32 days after a single dose or an equivalent period for the fractionated schedules. After fractionated irradiation the skin reactions developed 1–2 days later than after a single X-ray dose, and equivalent scoring periods (*e.g.* 12–34 days) were chosen by matching the leading edges of curves for reaction *versus* time (Denekamp, 1975).

### RESULTS

Fig. 1 illustrates dose-response curves

FIG. 1.—Average skin reactions over 23 days, as a function of X-ray dose. Each point shows the mean value for a group of 5 mice ± s.e. Solid symbols are for X-rays alone, irradiations in air (■) or $O_2$ (●). Open symbols denote MISO (670 mg/kg) given 30 min before irradiation in air (□) or $O_2$ (○). MISO sensitized the skin of air breathing and oxygen breathing mice to a common sensitivity.

* SER = (X-ray dose without MISO/X-ray dose with MISO) for equivalent damage in a population of hypoxic cells.

SER′ (observed SER) = (X-ray dose without MISO/X-ray dose with MISO) for a mixed population or for incompletely hypoxic cells.

FIG. 2.—Average skin reactions as a function of X-ray dose for 1, 2, 5 and 10 daily fractions irradiated in air. Control curves for X-rays alone (×——×) are shown, as well as curves for irradiations 30 min after 100 mg/kg MISO (○....○) or 670 mg/kg MISO (●- - -●). MISO sensitized the skin in each fractionation schedule.

FIG. 3.—Average skin reactions for 1 and 10 daily fractions with irradiations in $O_2$. Data are shown for X-rays alone (×——×), 100 mg/kg MISO (○) or 670 mg/kg MISO (●) given 30 min before irradiation. Sensitization was seen with single doses but not with 10 fractions.

for skin irradiated with single doses in air or $O_2$, either without drug or 30 min after administering 670 mg/kg MISO. A significant enhancement of the skin reaction was obtained if mice were irradiated in $O_2$ instead of air (dose-modifying factor (DMF) = 1·2). The skin response was further enhanced by MISO to give a common sensitivity whether in air or $O_2$. The SER′ values are therefore higher for animals in air than for those in $O_2$. SERs from these data are quoted in Table I.

Fig. 2 summarizes single-dose and fractionation schedules for irradiation in air. For all the schedules tested, MISO significantly enhanced the radiation response; SERs are given in Table I. The degree of sensitization for a particular drug dose was similar whether the radiation was given as a large single dose or as many small fractions.

Fig. 3 shows data from similar single-dose and 10-fraction experiments after irradiation in $O_2$. MISO sensitized the skin to single doses of X-rays (left panel)

TABLE I.—*Sensitizer enhancement ratios for mouse skin*

| No. of X-ray fractions | Air MISO dose (mg/kg) 100 | Air MISO dose (mg/kg) 670 | Oxygen MISO dose (mg/kg) 100 | Oxygen MISO dose (mg/kg) 670 |
|---|---|---|---|---|
| 1 | 1·18±0·05* | 1·2±0·06*<br>1·3±0·04† | 1·05±0·03* | 1·06±0·03 |
| 2 (1d) | — | 1·21±0·02* | — | — |
| 5 (4d) | — | 1·24±0·03* | | |
| 10 (11d) | 1·08+0·02* | 1·21±0·03*<br>1·17±0·03† | 1·0 | 1·0 |

SER′ measured at skin reaction level 1·0±s.e. (from fractional dose errors using envelopes through error bars on dose–response curves).

* Values from first experiment; data in Figs. 1 & 2.

† Values from a repeat experiment; data not shown.

FIG. 4.—Single-dose and 10-fraction curves for X-rays alone (×) or 670 mg/kg MISO given 5 min *after* each radiation dose (○). The upper panel shows data for irradiation in air and the lower panel shows data for irradiation in normobaric $O_2$. No significant cytotoxic killing by MISO was detected.

but not to 10 small fractions (right panel).

When MISO is given *before* irradiation, radiosensitization is measured together with any cytotoxicity due to its metabolic reduction in hypoxic cells to a toxic product (Hall & Roizin-Towle, 1975; Sutherland, 1974). In order to measure this cytotoxicity separately from radiosensitization, the MISO can be given *after* irradiation (Denekamp, 1978). Fig. 4 illustrates data from "post effect" experiments in skin. MISO was given 5 min *after* irradiation with single doses or 10 fractions, to mice breathing air or $O_2$. For irradiation in air (top panel) there was no evidence of cytotoxic killing in single doses, but the 10-fraction dose–response curve with MISO lies slightly to the left of the curve for X-rays alone (DMF = $1{\cdot}05 \pm 0{\cdot}03$). This shift is not significant, but suggests either a small amount of cell killing or an interference with X-ray repair processes in the presence of MISO (see Discussion). Single-dose irradiation in $O_2$ (Fig. 4, bottom panel) with MISO after X-rays produced a similar DMF (1·0 to $1{\cdot}08 \pm 0{\cdot}07$) again not significant. For 10 fractions in $O_2$ there was slight radioprotection when MISO was given after each irradiation.

MISO has been shown to cause a dose-dependent decrease in the body temperature of rodents (Johnson *et al.*, 1980; Hirst, personal communication). In the present experiments a reduction in rectal temperature was seen over the period when mice would normally have been irradiated (*i.e.* up to 60 min after injection). MISO at 100 mg/kg caused only a small drop from 38·8 to 37·8°C, but 670 mg/kg MISO caused a more extensive fall in body temperature, to 34°C by 45 min after a single injection, and similarly to 35°C after the last of 10 fractions. Thus the core temperature of MISO-treated mice was 1–5°C below normal body temperature at the time of irradiation, and the foot-skin temperatures (which were not measured) may have been even lower.

### DISCUSSION

These data clearly demonstrate that the skin of unanaesthetized mice is sensitized by MISO, even at a drug dose as low as 100 mg/kg and radiation fractions as low as 5 Gy (Fig. 2). The data have been analysed to determine whether cells are at an intermediate $O_2$ tension, or a small proportion of cells are at a very low $O_2$ tension, *e.g.* those over a critical distance (100–150 $\mu$M) from the nearest blood vessel.

Fig. 5 indicates how these two possibilities might be distinguished. In the top panel, survival curves are shown for two model populations; treated in ambient conditions, made completely hypoxic, or

FIG. 5.—Computer simulations of surviving fraction and SER's as a function of X-ray dose for mixed populations of oxic and hypoxic cells or for uniform populations at differing oxygenation. The assumed values of $D_0$ were 1·35 Gy for oxic, 3·64 Gy for hypoxic and 1·89 Gy for cells at intermediate $O_2$ tension. Panel A illustrates the lack of sensitization below 10 Gy for a mixed population. Panel B illustrates sensitization at all dose levels when all cells are at an intermediate $O_2$ tension.

FIG. 6.—SER' values as a function of X-ray dose for mice treated with 100 mg/kg (○) or 670 mg/kg MISO (●) before irradiation in air. The SER' values have been obtained at different dose levels (corresponding to skin reaction levels between 0·5 and 1·75) from each set of data. For the higher MISO dose the SER' values are constant at all X-ray doses, indicating a uniform mild hypoxia in skin. The error bars represent ± s.e.

sensitized by MISO. Fig. 5A shows the calculated effect of sensitizer on a mixed population of oxic and hypoxic cells (1% and 5% hypoxic fractions). Virtually no effect of the sensitizer is seen at X-ray doses below 10 Gy, because the response is dominated by the oxic cells. SER' values calculated from these hypothetical curves are plotted as a function of X-ray dose in the lower panels of Fig. 5. As the radiation dose increases the observed SER' progressively increases from unity to the maximum SER for fully hypoxic cells. By contrast, if *all* cells are at an intermediate $O_2$ tension, as in Fig. 5B, sensitization by MISO is the same at *all* radiation dose levels, and is always smaller than the effect on fully hypoxic cells. This would lead to a constant SER' *versus* dose-per-fraction curve, as illustrated in the lower panel of Fig. 5B. Thus an analysis of SER' values as a function of radiation dose should allow us to distinguish between the two possibilities. This approach is similar to that adopted by Hendry (1979) for comparing measured OER values for normal tissues treated in $O_2$, anoxia or air.

Fig. 6 summarises the SER' values derived from the data in Fig. 2 for mice irradiated in air. A wide range of X-ray dose per fraction can be covered, both for different levels of reaction within one fractionation scheme, and because single dose and fractionated data are available. It is clear that with 670 mg/kg MISO the SER' is constant over a wide range of X-ray dose. The SER' values do not vary significantly from 5 Gy to 32 Gy per fraction. The data from the low drug dose are more equivocal. Some sensitization *is* observed at 5–6 Gy per fraction, but there is a tendency to higher values at 25–32 Gy, though the error bars overlap.

This analysis demonstrates that the response of unanaesthetized mouse skin is more consistent with a uniform population of slightly hypoxic cells than with simply a very small fraction of severely hypoxic cells. Other workers (*e.g.* Dixon, 1967; Withers, 1967) have also concluded that murine normal tissues have a uniform level of mild hypoxia rather than a small proportion of severely hypoxic cells. This view is supported by a variety of studies which were reviewed by Hendry (1979), comparing anoxic, aerobic and fully oxygenated irradiations. The present data (Fig. 3) demonstrate some MISO sensitization even in $O_2$-breathing mice; this indicates that pure $O_2$ at atmospheric pressure does not fully sensitize all cells in mouse skin. Unexpectedly, 670 mg/kg MISO was more effective in sensitizing skin of air-breathing mice than changing the inspired gas from air to pure normobaric $O_2$, despite MISO being less efficient than $O_2$ on a molar basis (Adams, 1977; Suit *et al.*, 1981). Sensitization was obtained with low and high doses of MISO for single X-ray treatments, but not with 10 fractions in $O_2$. This would suggest that pure $O_2$ increases the $O_2$ tension in *most* of the skin cells, leaving a somewhat resistant subpopulation that can only be detected at high dose levels, as in Fig. 5A. This could also explain the absence of MISO sensitization in skin clone experiments for irradiation in $O_2$ (Denekamp *et al.*, 1974) where the dose range was < 20 Gy.

The experimental normal tissues in which MISO sensitization of X-ray damage has been tested in rodents are summarized in Table II. Three quarters of the studies show a small SER′. Published information for fractionated treatments in normal tissues is very sparse (Suit *et al.*, 1981) but this information is clearly needed to interpret the relevance of mouse data to current clinical trials with repeated small X-ray doses. No MISO sensitization of human skin under ambient conditions was observed in the early studies of Dische *et al.* (1976). Similarly, no enhanced normal-tissue damage has been found in the first 200 patients treated with MISO at Mount Vernon Hospital (Dische *et al.*, 1979). The only clinical trial in which enhanced normal-tissue reactions have so far been reported is the Italian study (Arcangeli & Nervi, 1980) for irradiations of oropharyngeal

TABLE II.—*Summary of published single-dose studies for MISO sensitization of normal tissues*

| Tissue | MISO dose (mg/Kg) | SER′ | Gas phase during irradiation | Author |
|---|---|---|---|---|
| Skin (foot)† | 100 | 1·0–1·1 | $O_2$ | Stewart *et al.* |
| | 100 | 1·1–1·2 | air | (present data) |
| | 670 | 1·0–1·1 | $O_2$ | |
| | 670 | 1·2–1·3 | air | |
| Skin (leg)* | 300 | 1·0–1·3 | air | Brown, 1975 |
| | 1000 | 1·0–1·3 | air | Brown, 1975 |
| Skin (foot) | 200 | 1·2 | air | Yuhas *et al.*, 1977 |
| Skin (thigh)‡ | 200 | 1·1 | air | Yuhas *et al.*, 1977 |
| Skin (thigh) | 300 | 1·5 | air | Suit *et al.*, 1981 |
| Testis | 1000 | 1·3 | air | Suzuki *et al.*, 1977 |
| Tail necrosis | 1000 | 1·1 | $O_2$ | Hendry, 1978 |
| | 1000 | 1·5 | air | Hendry, 1978 |
| Tibial cartilage | 500 | 1·3 | air | Gonzalez & Breur, 1978 |
| Spinal cord* | 200 | 1·3 | air | Yuhas, 1979 |
| Oesophagus* | 1000 | 1·5 | $O_2$ | Hornsey & Field, 1979 |
| | 1000 | 1·6–1·8 | air | |
| Marrow | 400–800 | 0·8–1·0 | air | Yuhas *et al.*, 1977 |
| Marrow | 1000 | 1·0 | air | Hendry, 1978 |
| Intestine | 1000 | 1·0 | air | Hendry, 1978 |
| Brain* | 1000 | 1·0 | air | Field & Morris, 1981 |
| Spinal cord | 1000 | 1·0 | air | Travis *et al.*, 1982 |

* Irradiations under anaesthesia.

† Includes IF, 2F, 5F & 10F data.

‡ 5F data.

sites. However, increased clinical skin and bowel reactions were found after irradiation in hyperbaric $O_2$ (Dische, 1979; Henk *et al.*, 1977) which would again be consistent with uniform mild hypoxia rather than a small fraction of acutely hypoxic cells.

The relevance of the present mouse results to the response of human tissues in patients undergoing radiotherapy clearly depends on whether tissue oxygenation in a rodent resembles that in humans. Mouse skin is thinner than human skin, and the hair follicles at least are known to derive some of their $O_2$ from the ambient gas phase, rather than through the vasculature (Potten & Howard, 1969). Skin is also a major thermoregulatory tissue, and may be grossly influenced by ambient experimental conditions. However, the present results (Table I) and others in the literature (Hendry, 1979; Denekamp *et al.*, 1981) indicate that many normal-tissue cells may be closer to radiobiological hypoxia than is often supposed. This might make them easy to protect against radiation injury, but it also means that they may be sensitized by radio-sensitizers like MISO. Such a uniform low $O_2$ tension would imply that most of the cells in certain tissues are below the venous $O_2$ tension, or that the $O_2$ K value *in vivo* differs from that *in vitro* (Withers, 1967; Hendry, 1979; Denekamp *et al.*, 1981).

Sensitization of normal tissue by MISO should be taken into account in assessing its therapeutic value for experimental tumours. A therapeutic benefit will only exist if there is more sensitization in tumours than in normal tissues. Early experiments with skin irradiation in $O_2$ indicated no significant MISO sensitization at doses below 25 Gy (Denekamp *et al.*, 1974). We have therefore published therapeutic comparisons of MISO-treated tumours with those receiving X-rays alone on the basis of no skin-damage enhancement (Denekamp & Harris, 1976; Denekamp *et al.*, 1976). These comparisons show a slight decrease in the benefit of MISO compared with the present sets of skin data. The single-dose sensitization in tumours is however much larger than for skin (*e.g.* SER′ 1·7–2·4). Therefore there is still a big therapeutic gain for murine tumours treated with single doses of X-rays +MISO which is, however, less marked for fractionated treatments.

The present single-dose and fractionated data can be compared to investigate the effects of MISO on the repair capacity of mouse skin. A reduced ability to repair *potentially* lethal radiation damage has been demonstrated *in vitro* and *in vivo* with MISO given before or after irradiation of both oxic and hypoxic cells (Guichard *et al.*, 1979; Nakatsugawa & Sugahara, 1980; Sakamoto & Aritake, 1981). Repair of radiation damage has been estimated from dose–response curves in the present series of experiments (Figs. 2 & 3) by comparing the doses required in single and fractionated treatments to give the same skin reaction. These experiments do not allow us to distinguish between repair of sublethal and potentially lethal damage (PLD). Thus we are measuring total repaired damage, including any PLD there might be. Repair increments $(D_N - D_1/N - 1)$* have been calculated for X-rays alone, MISO given before X-rays and MISO after X-rays. In Fig. 7 the recovered dose per interval is plotted as a function of the X-ray dose per fraction. Repair increments calculated from dose curves for irradiation in air and $O_2$ (Figs 2 & 3) are shown as points, with a solid line representing previously published values for mouse skin (Fowler *et al.*, 1972, 1974; Denekamp, 1973; Denekamp & Harris, 1976; Douglas & Fowler, 1976). The present data for X-rays alone, whether in air or $O_2$, agree well with published values. MISO given before or after

* D = X-ray dose; N = number of fractions.

FIG. 7.—Recovered dose ($D_N$-$D_1$/N-1) as a function of X-ray dose/fraction. Errors represent ±s.e. derived as fractional dose errors from envelope errors on dose–response curves. Repair values for mice treated with X-rays only or with MISO *after* irradiation, fall on the solid line, representing previously published data (see text). Mice sensitized by MISO given *before* X-rays had a reduced repair capacity, particularly at high X-ray doses. (In air: ×, X-ray only. ●, MISO pre X. ■, MISO post X. In $O_2$: +, X-ray only. ○, MISO pre X. □, MISO post X.)

irradiation caused an apparent reduction in repair, particularly at high doses/fraction (*i.e.* in the 2-fraction experiment with MISO given before irradiation in air).

The reduction in body temperature associated with high doses of MISO could influence the skin response in several ways. A reduced core temperature could lead to considerable peripheral vasoconstriction, which would limit the available $O_2$; but this would also reduce $O_2$ consumption and hence increase the $O_2$ diffusion distances. These two opposing effects would respectively protect or sensitize the skin, but their relative magnitude is not known. If lower temperature in the MISO-treated mice is contributing to the apparent sensitization of mouse skin, it would imply that further $O_2$ diffusion in hypothermic tissues predominates over the effects of vasoconstriction. This temperature effect is likely to be more important for skin than for other normal tissues.

Significant radiosensitization by MISO has been seen in mouse skin with both high and low doses of MISO, and with doses of radiation as low as 5 Gy. Very little cytotoxicity has been found, but there is a small decrease in repair of radiation injury in the presence of MISO. These results appear to support the thesis of Hendry (1979) and others that rodent normal tissues contain a large proportion of cells at a critical intermediate $O_2$ tension which makes them marginally radioresistant.

We should like to thank Roche Products Ltd, Welwyn Garden City, for providing the misonidazole. We should also like to to thank Professor J. F. Fowler and Dr J. Hendry for their helpful criticism of this manuscript, Dr M. Joiner and Dr A. C. Begg for invaluable help with computer modelling for the hypoxic fraction estimates and the Cancer Research Campaign for financial support of this project.

### REFERENCES

ADAMS, G. E. (1977) Hypoxic cell sensitizers for radiotherapy. In *Cancer, A Comprehensive Treatise, Vol.* 6. (Ed. Becker). New York: Plenum Press. p. 181.

ARCANGELI, G. & NERVI, C. (1980) Misonidazole also radiosensitizes some normal tissue. *Br. J Radiol.*, **53,** 44.

BROWN, J. M. (1975) Selective radiosensitization of the hypoxic cells of mouse tumours with the nitroimidazoles, metronidazole and Ro 07-0582. *Radiat. Res.*, **64,** 633.

DENEKAMP, J. (1973) Changes in the rate of repopulation during multi-fraction irradiation of mouse skin. *Br. J. Radiol.*, **46,** 381.

DENEKAMP, J. (1975) Residual radiation damage in mouse skin 5 to 8 months after irradiation. *Radiology*, **115,** 191.

DENEKAMP, J. (1978) Cytotoxicity and radiosensitization in mouse and man. *Br. J. Radiol.*, **51,** 636.

DENEKAMP, J. & HARRIS, S. R. (1976) The response of a transplantable tumor to fractionated irradiation. I. X-rays and the hypoxic cell radiosensitizer Ro 07-0582. *Radiat. Res.*, **66,** 66.

DENEKAMP, J., HARRIS, S. R., MORRIS, C. & FIELD, S. B. (1976) The response of a transplantable tumor to fractionated irradiation. II. Fast neutrons. *Radiat. Res.*, **68,** 93.

DENEKAMP, J., MICHAEL, B. D. & HARRIS, S. R. (1974) Hypoxic cell radiosensitizers: Comparative test of some electron affinic compounds using epidermal cell survival *in vivo*. *Radiat. Res.*, **60,** 119.

DENEKAMP, J., MICHAEL, B. D., ROJAS, A. & STEWART, F. A. (1981) Thiol radioprotection *in vivo*: The critical role of tissue oxygen concentration. *Br. J. Radiol.*, **54,** 1112.

DISCHE, S. (1979) Hyperbaric oxygen: The Medical Research Council trials and their clinical significance. *Br. J. Radiol.*, **51,** 888.

DISCHE, S., GRAY, A. J. & ZANELLI, G. D. (1976) Clinical testing of the radiosensitizer Ro 07-0582. II. Radiosensitization of normal and hypoxic skin. *Clin. Radiol.*, **27**, 159.

DISCHE, S., SAUNDERS, M. I., FLOCKHART, I. R., LEE, M. E. & ANDERSON, P. (1979) Misonidazole: A drug for trial in radiotherapy and oncology. *Int. J. Radiat. Oncol. Biol. Phys.*, **5**, 851.

DIXON, B. (1967) The effect of radiation on the growth of vertebrae in the tails of rats. *Int. J. Radiat. Biol.*, **13**, 355.

DOUGLAS, B. G. & FOWLER, J. F. (1976) The effect of multiple small doses of X-rays on skin reactions in the mouse and a basic interpretation. *Radiat. Res.*, **66**, 401.

FIELD, S. B. & MORRIS, C. C. (1981) Does misonidazole enhance radiation injury to the central nervous system? *Br. J. Cancer*, **43**, 878.

FOWLER, J. F., DENEKAMP, J., DELAPEYRE, C., HARRIS, S. R. & SHELDON, P. W. (1974) Skin reactions in mice after multifraction X-irradiation. *Int. J. Radiat. Biol.*, **25**, 213.

FOWLER, J. F., DENEKAMP, J., PAGE, A. L. & BEGG, A. C. (1972) Fractionation with X-rays and neutrons in mice: Response of skin and C3H mammary tumours. *Br. J. Radiol.*, **45**, 237.

FOWLER, J. F., KRAGT, K., ELLIS, R. E., LINDOP, P. J. & BERRY, R. J. (1965) The effect of divided doses of 15 meV electrons on the skin response of mice. *Int. J. Radiat. Biol.*, **9**, 241.

GONZALES, D. G. & BREUR, K. (1978) Dose modifying effects of misonidazole on the radiation response of growing cartilage in mice. *Br. J. Cancer*, **37**, Suppl. III, 235.

GUICHARD, M., DE LANGEN-OMRI, F. & MALAISE, E. P. (1979) Influence of misonidazole on the radiosensitivity of a human melanoma in nude mice: Time-dependent increase in surviving fraction. *Int. J. Radiat. Oncol. Biol. Phys.*, **5**, 487.

HALL, E. J. & ROIZIN-TOWLE, L. (1975) Hypoxic sensitizers: Radiobiological studies at the cellular level. *Radiology*, **117**, 453.

HENDRY, J. H. (1978) Sensitization of hypoxic normal tissue. *Br. J. Cancer*, **37**, Suppl. III, 232.

HENDRY, J. H. (1979) Quantitation of the radiotherapeutic importance of naturally-hypoxic normal tissues from collated experiments with rodents using single doses. *Int. J. Radiat. Oncol. Biol. Phys.*, **5**, 971.

HENDRY, J. H. & SUTTON, M. L. (1978) Care with radiosensitizers. *Br. J. Radiol.*, **51**, 927.

HENK, J. M., KUNKLER, P. B., SMITH, C. W. (1977) Radiotherapy and hyperbaric oxygen in head and neck cancer. *Lancet*, ii, 101.

HORNSEY, S. & FIELD, S. B. (1979) The effects of single and fractionated doses of X-rays and neutrons on the oesophagus. *Int. J. Cancer*, **15**, 491.

JOHNSON, R. J. R., BURKE, T. R., DRECHSEL, R. & SUBJECK, J. R. (1980) Physiological changes which may result from *in vivo* experimental conditions. *Br. J. Cancer*, **41**, Suppl. IV, 106.

NAKATSUGAWA, S. & SUGAHARA, T. (1980) Inhibition of X-ray-induced potentially lethal damage (PLD) repair by cordycepin (3′-deoxyadenosine) and enhancement of its action by 2′-deoxycoformycin in Chinese hamster *hai* cells in the stationary phase *in vitro*. *Radiat. Res.*, **84**, 265.

POTTEN, C. S. & HOWARD, A. (1969) Radiation depigmentation of mouse hair: The influence of local tissue oxygen tension on radiosensitivity. *Radiat. Res.*, **38**, 65.

SAKAMOTO, K. & ARITAKE, S. (1981) Effects of misonidazole on tumor cell radiation sensitivity and potentially lethal damage repair *in vivo* and *in vitro*. *Eur. J. Cancer*, **17**, 825.

STEWART, F. A. & DENEKAMP, J. (1977) Sensitization of mouse skin to X-irradiation by moderate heating. *Radiology*, **113**, 195.

SUIT, H. D., MAIMONIS, P., MICHAELS, H. B. & SEDLACEK, R. (1981) Comparison of hyperbaric oxygen and misonidazole in fractionated irradiation of murine tumours. *Radiat. Res.*, **87**, 360.

SUTHERLAND, R. M. (1974) Selective chemotherapy of non-cycling cells in an *in vitro* tumour model. *Cancer Res.*, **34**, 3501.

SUZUKI, N., WITHERS, H. R. & HUNTER, N. (1977) Radiosensitization of mouse spermatogenic stem cells by Ro 07-0582. *Radiat. Res.*, **69**, 598.

TRAVIS, E. L., PARKINS, C. S., HOLMES, S. J. & DOWN, J. D. (1982) Effect of misonidazole on radiation injury in mouse spinal cord. *Br. J. Cancer*, **45**, 469.

WITHERS, H. R. (1967) The dose–survival relationships for irradiation of epithelial cells of mouse skin. *Br. J. Radiol.*, **40**, 187.

YUHAS, J. M. (1979) Misonidazole enhancement of acute and late radiation injury to the rat spinal cord. *Br. J. Cancer*, **40**, 161.

YUHAS, J. M., YURCONIC, M., KLIGERMAN, M. M., WEST, G. & PETERSON, D. F. (1977) Combined use of radioprotective and radiosensitizing drugs in experimental radiotherapy. *Radiat. Res.*, **70** 433.